# Correlation between genetic polymorphism of matrix metalloproteinase-9 in patients with coronary artery disease and cardiac remodeling

**DOI:** 10.12669/pjms.313.7229

**Published:** 2015

**Authors:** Qibin Yu, Hanmei Li, Linlin Li, Shaoye Wang, Yongbo Wu

**Affiliations:** 1 Qibin Yu, Department of Cardiosurgery, Fuwai Hospital, Chinese Academy of Medical Science and Peking Union Medical College, Peking 100037, China; 2 Hanmei Li, Department of Cardiosurgery, Fuwai Hospital, Chinese Academy of Medical Science and Peking Union Medical College, Peking 100037, China; 3 Linlin Li, Department of Cardiosurgery, Fuwai Hospital, Chinese Academy of Medical Science and Peking Union Medical College, Peking 100037, China; 4 Shaoye Wang, Department of Cardiosurgery, Fuwai Hospital, Chinese Academy of Medical Science and Peking Union Medical College, Peking 100037, China; 5 Yongbo Wu, Department of Cardiosurgery, Fuwai Hospital, Chinese Academy of Medical Science and Peking Union Medical College, Peking 100037, China

**Keywords:** Coronary artery disease, Matrix metalloproteinase-9, Genetic polymorphism, Cardiac remodeling

## Abstract

**Objective:**

To explore the correlation between genetic polymorphism of matrix metalloproteinase-9 (MMP-9) in patients with coronary artery disease (CAD) and cardiac remodeling.

**Methods:**

A total of 272 subjects who received coronary angiography in our hospital from July 2008 to September 2013 were selected, including 172 CAD patients (CAD group) and another 100 ones (control group). Both groups were subjected to MMP-9 and ultrasonic detections to determine vascular remodeling and atherosclerotic plaques. C1562G polymorphism of MMP-9 gene was detected, and correlation with vascular remodeling and atherosclerotic plaque was analyzed.

**Results:**

Serum MMP-9 level of CAD group (330.87±50.39 ng/ml) was significantly higher than that of control group (134.87±34.02 ng/ml) (P<0.05). Compared with control group, CAD group had significantly higher intima-media thickness, and significantly lower systolic peak velocity, mean systolic velocity and end-diastolic velocity (P<0.05). Total area of stenotic blood vessels was 67.34±22.98 mm^2^, while that of control blood vessels was 64.00±20.83 mm^2^. G/G, G/C and C/C genotype frequencies of MMP-9 differed significantly in the two groups (P<0.05). G and C allele frequencies of CAD group (70.9% and 29.1%) were significantly different from those of control group (50.0% and 50.0%) (P<0.05). G/G, G/C and C/C genotypes were manifested as lipid-rich, fibrous and calcified or ulcerated plaques respectively. Total area of stenotic blood vessels of G/G genotype significantly exceeded those of G/C and C/C genotypes (P<0.05), whereas the latter two had no significant differences.

**Conclusion:**

CAD promoted 1562C-G transformation of MMP-9 gene into genetic polymorphism, thus facilitating arterial remodeling and increasing unstable atherosclerotic plaques.

## INTRODUCTION

Coronary artery disease (CAD), as a common, terminal heart disease severely threatening human health, has high disabling and morality rates. Recently, the incidence of CAD has been skyrocketing worldwide.^1^,^2^ Inflammatory response plays a key role in atherosclerosis, and the formation, growth and rupture of atherosclerotic plaques are closely associated with various growth factors, cytokines, mononuclear macrophages, lymphocytes and adhesion molecules. Meanwhile, cardiac remodeling is also predominantly controlled by changes, structures and compositions of plaques.^3^-^5^ Intravascular ultrasound can accurately disclose size and morphology of coronary lumen, anatomical structure of vessel wall, as well as characteristics of plaques. In CAD patients, carotid and femoral arteries are prone to atherosclerosis early, initiating further progression.^6^

As a family of important proteases that can digest extracellular matrix (ECM), matrix metalloproteinases (MMPs) participate in ECM degradation and reconstruction, and thus unstablize plaques eventually.^7^,^8^ MMP-9, which is an essential member of the MMPs family, participates in ECM degradation and significantly regulates inflammatory response. Besides, MMP-9 is involved in tissue reconstruction by decomposing almost all constituents besides ECM.^9^ There is C-G polymorphism at -1,562 in the promoter region of MMP-9, and this mutation site weakens the inhibition of gene transcription.^10^

MMP-9 C1562G polymorphism may be a risk factor of angina, and is related with ischemic cardiomyopathy and in-stent restenosis. However, its correlation with CAS remains unclear hitherto.^11^,^12^ In this study, the correlation between genetic polymorphism of MMP-9 in CAD patients and cardiac remodeling was analyzed.

## METHODS

### Subjects

A total of 272 subjects who received coronary angiography in our hospital from July 2008 to September 2013 were selected. This study was approved by the ethic committee of our hospital, and written consent has been obtained from all subjects.

### Inclusion criteria

20-80 years old; secondary hypertension, liver and kidney diseases, infections, connective tissue diseases and malignant tumors were excluded by inquiry of medical records, physical examination, electrocardiogram and laboratory test; without blood relations.

### Diagnostic criteria for 172 CAD patients (CAD group)

Coronary angiograms were checked by two experienced physicians, and those with at least one of the left anterior descending branch (LAD), the left circumflex branch (LCX) and the right coronary artery (RCA) being stenosis ≥50% were considered positive. This group comprised 100 males and 72 females, aged 34-79 years old (average: 62.34±3.11). Coronary angiography confirmed that there were 102 cases of single-vessel disease, 43 cases of double-vessel disease and 27 cases of three-vessel disease. As to disease types, there were 72 cases of myocardial infarction and 100 cases of unstable angina. The control group (n=100) had negative coronary angiograms, comprising 56 males and 44 females aged 32-80 years old (62.45±3.09). The two groups had similar age, gender, body mass index (BMI), as well as levels of fasting blood glucose (FBG), total cholesterol (TC) and triglyceride (TG) (P>0.05) (Table-I).

**Table-I:** Baseline clinical data.

Index	Control group (n=100)	CAD group (n=172)	t or χ^2^	P
Gender (male/female)	56/44	100/72	0.234	>0.05
Age (years old)	62.45±3.09	62.34±3.11	0.183	>0.05
BMI (kg/m^2^)	22.76±5.39	22.98±4.83	0.122	>0.05
FBG (mmol/L)	5.67±1.33	5.72±1.63	0.199	>0.05
TC (mmol/L)	4.57±0.87	4.64±0.63	0.142	>0.05
TG (mmol/L)	1.45±0.24	1.55±0.45	0.872	>0.05

### Detection of serum MMP-9

Fasting cubital venous blood (5 ml) was collected in the morning and detected within two hour. Blood sample was centrifuged at 1000 r/minutes for 15 min, from which the supernatant was collected and stored in a -20°C refrigerator. MMP-9 levels were detected by using double antibody sandwich ABC-ELISA according the instructions of kit (Shanghai Senxiong Technology Industry Co., Ltd.). FBG and blood lipid levels were measured at the same time.

### Ultrasonic detection

Atherosclerotic plaques were defined when intima-media thickness (IMT) of posterior carotid artery wall≥ 1.2 mm, and classified into lipid-rich plaque, fibrous plaque, calcified plaque and ulcerated plaque according to two-dimensional ultrasonography. Lipid-rich plaque, which originated from intimal lipid deposition, was ultrasonically disclosed as hypoechoic, homogeneous thickening of the intima. Fibrous plaque referred to continuous fibrous cap contours on the surface, with local, homogeneous hyperecho. Calcified plaque referred to fibrous, calcified regions with enhanced echoes, accompanied by posterior acoustic shadow or obvious echo attenuation. Ulcerated plaque had irregular surface and hypoecho at the edges. Meanwhile, systolic peak velocity (SPV), mean systolic velocity (MSV) and end-diastolic velocity (EDV) were measured by carotid artery blood-flow frequency spectrum.

The mean of normal blood vessel cross-sectional areas in the proximal and distal lesions was used as the reference one, and the cross-sectional areas of stenotic blood vessels were also determined. Since the control group hardly suffered from vascular remodeling or plaques, this detection was only applied to the CAD group.

### Analysis of MMP-9 genetic polymorphism

Venous blood (2-3 ml) was collected and subjected to routine anticoagulation, from which DNA was extracted with phenol by using a kit (TaKaRa Biotechnology (Dalian) Co., Ltd.). Afterwards, DNA was diluted to 20 μg/L and stored at 4°C prior to use. Then PCR-RALP was performed. By using primers for MMP-9 designed by Sangon Biotech (Shanghai) Co., Ltd. (upstream: 5-GCCTGGCACArAGTAGGCCC-3; downstream: 5-CTTCCTAGCCAGCCGGC-3; internal reference: actin), PCR was conducted at 55°C for annealing and for 30 s of extension, 35 cycles in total. The amplified product was subjected to polyacrylamide gel electrophoresis for polymorphism analysis.

### Statistical analysis

All data were analyzed by SPSS 13.0. The categorical data were expressed as mean ± standard deviation (x ± s), and inter-group comparisons were performed with t test. The numerical data were compared by Chi-square test, and subjected to nonparametric rank correlation test. P<0.05 was considered statistically significant.

## RESULTS

### MMP-9 expression levels

Serum MMP-9 level of CAD group (330.87±50.39 ng/ml) was significantly higher than that of control group (134.87±34.02 ng/ml) (P<0.05) (Table-II).

**Table-II:** MMP-9 expression levels (ng/ml, x±s).

Group	Case number (n)	MMP-9
CAD group	172	330.87±50.39
Control group	100	134.87±34.02
t		12.987
P		<0.05

### Ultrasonic indices

Compared with control group, CAD group had significantly higher IMT, and significantly lower SPV, MSV and EDV (P<0.05) (Table-III). CAD group consisted of 26 cases of lipid-rich plaque, 100 cases of fibrous plaque, 24 cases of calcified plaque and 22 cases of ulcerated plaque.

**Table-III:** Ultrasonic indices (x±s).

Group	Case number (n)	IMT (mm)	SPV (m/s)	MSV (m/s)	EDV (m/s)
CAD group	172	0.11±0.02	63.78±11.44	44.78±11.98	17.37±7.39
Control group	100	0.07±0.01	77.65±19.37	50.82±12.87	25.38±8.79
t		6.480	7.992	5.398	9.334
P		<0.05	<0.05	<0.05	<0.05

### Vascular remodeling results

The total area of stenotic blood vessels was 67.34±22.98 mm^2^, while that of control blood vessels was 64.00±20.83 mm^2^.

### MMP-9 genotype distribution

The G/G, G/C and C/C genotype frequencies of MMP-9 differed significantly in the two groups (P<0.05). The G and C allele frequencies of CAD group (70.9% and 29.1%) were significantly different from those of control group (50.0% and 50.0%) (P<0.05) (Table-IV).

**Table-IV:** MMP-9 genotype and allele frequencies (n)

Group	Case No.	Genotype frequency			Allele frequency	
		G/G	G/C	C/C	G	C
CAD group	172	51 (29.7%)	89 (51.7%)	32 (18.6%)	122 (70.9%)	50 (29.1%)
Control group	100	18 (18.0%)	30 (30.0%)	52 (52.0%)	50 (50.0%)	50 (50.0%)
χ^2^		6.872	7.093			
P		<0.05	<0.05			

G/G, G/C and C/C genotypes were manifested as lipid-rich plaque, fibrous plaque and calcified or ulcerated plaque respectively (Table-V). The total area of stenotic blood vessels of G/G genotype significantly exceeded those of G/C and C/C genotypes (P<0.05), whereas the latter two genotypes had no significant differences (Table-VI).

**Table-V:** Plaque types of CAD patients with different genotypes (n).

Genotype	Case No.	Lipid-rich plaque (n=26)	Fibrous plaque (n=100)	Calcified plaque (n=24)	Ulcerated plaque (n=22)
G/G	51	25	17	2	6
G/C	89	1	81	7	0
C/C	32	0	2	15	16

**Table-VI:** Vascular remodeling results of CAD patients with different genotypes (mm^2^, x±s)

Genotype	Case number (n)	Total area of stenotic blood vessels	Total area of control blood vessels
G/G	51	71.38±15.39	63.78±14.98
G/C	89	65.38±12.77	64.03±14.38
C/C	32	63.09±12.76	64.22±12.09
F		8.787	0.457
P		<0.05	>0.05

## DISCUSSION

With improved standard of living and population aging, CAD has endangered numerous people.^13^ In addition to direct damages, CAD also injures all target organs in human body and leads to atherosclerosis. Changes of the intima are now measured based on IMT by using high-frequency ultrasonic probe. Carotid artery, as one of the most involved blood vessels upon atherosclerosis, indirectly reflects the degrees of coronary arterial and systemic atherosclerosis.^14^ IMT indicates the thickening of arterial wall, which is an early sign of atherosclerosis. Increase of IMT by every 0.1 mm elevates the risk of CAD. Moreover, with shallow location and linear shape, carotid artery allows repeated measurements and ultrasonic observation of arterial IMT.^15^ In this study, compared with control group, CAD group had significantly higher IMT, and significantly lower SPV, MSV and EDV (P<0.05). The intima responds to local blood flow and changes of vascular wall tension through hyperplasia and fibrocyte hypertrophy. Manifested as crescent-shaped deposition of intimal ECM and smooth muscle cells, early atherosclerosis is ultrasonically disclosed as integral intima, so increase of IMT is not the sole outcome.

MMPs, which are Zn^2+^-dependent proteases, are associated with degradation of collagen and play crucial roles in maintenance and reconstruction of vascular wall.^16^ MMP-9 can destruct vascular wall by efficiently degrading Type IV collagen, the high expression of which induces vascular remodeling and arteriosclerosis. In the meantime, decrease in the thickness of fibrous cap at the injury site raises the risk of plaque rupture and may result in death by inducing CAD.^17^ Biologically speaking, MMP-9 exerts catalytic action depending on the active center Zn^2+^. As suggested by DNA sequences, MMP-9 shares homology with collagenase, which is secreted as zymogen that can be activated by proteases or organic mercury compounds. The activity of MMP-9 can be inhibited by corresponding inhibitors.^18^ In this study, the serum MMP-9 levels of CAD group (330.87±50.39 ng/ml) was significantly higher than that of control group (134.87±34.02 ng/ml) (P<0.05). Currently, degree of coronary arterial stenosis has been positively correlated with MMP-9. CAD patients have higher serum MMP-9 levels than those of normal people, inferring that MMP-9 is related with rupture of atherosclerotic plaques, coronary occlusion and myocardial ischemic injury.

Since CAD patients usually have inconsistent degrees of atherosclerosis in different arteries or in different segment of the same artery, assessing atherosclerosis with quantitative and qualitative methods is more accurate. Ulcerated plaque and calcified plaque are stable, whereas the others are unstable.^19^ Unstable plaques are pathologically typified by large lipid cores and thin fibrous caps, but stable plaques are mainly manifested as concentric stenosis, smooth boundary and free of filling defect. The CAD group herein comprised 26 cases of lipid-rich plaque, 100 cases of fibrous plaque, 24 cases of calcified plaque and 22 cases of ulcerated plaque, suggesting that CAD mainly involved unstable plaques. Hence, it is possible to indirectly study CAD by observing the stability of carotid arterial plaques.

Vascular remodeling is a compensatory response to external environment changes such as injury, ischemia and anoxia, hemodynamic resistance, arterial injury and cell proliferation, mainly manifested as increased cross-sectional area of the external elastic membrane.^20^ In this study, the total area of stenotic blood vessels was 67.34±22.98 mm^2^, while that of control blood vessels was 64.00±20.83 mm^2^. Therefore, the lumen area did not evidently decrease even upon severe stenosis, which provided sufficient blood for distal lesions and prevented or postponed adverse cardiac events.

With the whole length of 7.7 kb, human MMP-9 gene has ten sequence variations in the promoter region and 13 exons, of which C-1562T, as the common mutation site with C replaced by G, binds transcriptional repressor proteins. Upon C-G mutation, DNA- protein interaction is eliminated, thereby affecting gene transcription to produce lowly active C/C genotype and highly active C/G and G/G ones.^21^ The G/G, G/C and C/C genotype frequencies of MMP-9 herein differed significantly in the two groups (P<0.05). The G and C allele frequencies of CAD group (70.9% and 29.1%) were significantly different from those of control group (50.0% and 50.0%) (P<0.05). The results indicated C-G transformation enhanced the activity of gene transcription, participated in ECM degradation, invasion of inflammatory cells and plaque rupture during atherosclerosis, and finally facilitated the onset of CAD and other acute vascular events.

It is now well-established that MMP-9 expression was associated with the progression of atherosclerosis, and that MMP-9 genetic polymorphism was significantly correlated with acute myocardial infarction but not degree of coronary artery stenosis.^9^ Shevchenko et al. reported that the lesion area of G allele carriers significantly exceeded that of C allele ones, and that the stenotic rate of coronary artery was significantly correlated with GG genotype. In addition, G allele carriers were significantly more prone to myocardial infarction.^12^ In patients with acute myocardial infarction and unstable angina, the ones suffering from plaque rupture have higher MMP-9 levels than those without, and the patients have more obvious 1562C-G polymorphism than the controls do. Moreover, the patients who die of atherosclerosis are typified in large lipid cores and vigorous vascular remodeling.^17^ In this study, G/G, G/C and C/C genotypes were manifested as lipid-rich plaque, fibrous plaque and calcified or ulcerated plaque respectively. The total area of stenotic blood vessels of G/G genotype significantly exceeded those of G/C and C/C genotypes (P<0.05), whereas the latter two genotypes had no significant differences. Accordingly, G/G genotype could accelerate the degradation of fibrous caps and unstabilize plaques. Furthermore, different genotypes had various plaque types, probably because highly expressed genotypes were related with the progression of atherosclerosis.

In summary, CAD patients were accompanied by increase of MMP-9 expression and abnormal carotid ultrasound. CAD was conducive to 1562C-G transformation of MMP-9 gene into genetic polymorphism, thus promoting arterial remodeling and increasing unstable atherosclerotic plaques.
